# Research Advances in Therapeutic Strategies and Drug Delivery Systems for Pathological Scars

**DOI:** 10.3390/pharmaceutics18020148

**Published:** 2026-01-23

**Authors:** Yuxin Shi, Ling Li

**Affiliations:** 1 School of Medicine, Chongqing University, Chongqing 400030, China; 202337021065t@stu.cqu.edu.cn; 2 State Key Laboratory of Trauma and Chemical Poisoning, Institue of Burn Research, Army Medical University, Chongqing 400038, China

**Keywords:** pathological scars, biomaterial-based drug delivery, microneedles, scarless wound healing, targeted therapy

## Abstract

Pathological scars are fibrotic lesions that result from aberrant wound healing following tissue injury, such as burns. They are frequently associated with disfigurement and dysfunction, thereby severely impairing the quality of life of affected patients. Current clinical treatments, including surgery, laser therapy, and corticosteroid injections, are often characterized by limited efficacy, high recurrence rates, and undesirable side effects, including skin atrophy. Furthermore, the dense structure and excessive extracellular matrix (ECM) deposition in scar tissue present a significant barrier to effective drug penetration, thereby further limiting therapeutic efficacy. In recent years, biomaterial-based drug delivery systems, which integrate sustained drug release with minimally invasive transdermal technologies, have emerged as a promising strategy to overcome the limitations of traditional therapies. This review systematically outlines the pathogenesis and molecular mechanisms of pathological scars, summarizes established and emerging treatments, and highlights the application strategies and future prospects of novel biomaterial-based drug delivery systems for managing this condition.

## 1. Introduction

Severe burn injuries frequently result in pathological scars, which affect approximately 70% of survivors and lead to disfigurement, pain, functional impairment, and psychological distress [1,2]. Globally, an estimated 100 million people are affected by scar-related conditions [3], imposing a substantial socioeconomic burden reflected by annual expenditures exceeding $10.3 billion in the UK alone, with even higher costs in the United States [4]. Pathological scars, primarily categorized as hypertrophic scars and keloids, are fibrotic disorders caused by aberrant wound healing [1]. While hypertrophic scars remain confined to the original wound boundaries, keloids are characterized by invasive growth and a high propensity for recurrence following surgical intervention [5]. The efficacy of current clinical treatments, such as corticosteroid injections, is often limited. This limitation is primarily attributed to the absence of hair follicles and other skin appendages in scar tissue, which impedes deep drug penetration and frequently leads to suboptimal outcomes and recurrence [6]. Moreover, these interventions can induce adverse effects, such as skin atrophy and hypopigmentation [7]. Accordingly, there is an urgent clinical need for a therapeutic strategy that can overcome the physical barrier of scars, enable deep, targeted, and sustained drug delivery, possess both safety and durability, and fundamentally repair pathological scars. Since none of the existing therapeutic methods can meet this demand, it underscores the imperative need for the development of novel and efficient drug delivery systems.

In recent years, nanomaterials and microneedles have gained prominence as core components of advanced drug delivery systems due to their distinct advantages. The small size, high surface-to-volume ratio, and functionalizable surfaces of nanomaterials facilitate sustained and targeted drug release, which can reduce dosage frequency and enhance therapeutic efficacy [8,9]. Similarly, microneedles provide a minimally invasive and painless means of bypassing the stratum corneum, enabling precise local drug delivery [10]; several microneedle-based devices have already progressed to clinical trials [11]. Furthermore, advances in biomaterial design have enabled the overcoming of biological barriers, allowing for controlled drug release and establishing a robust platform for therapeutic applications [12,13]. These systems not only offer the potential to target key signaling pathways in scar formation (e.g., Transforming Growth Factor-beta) but also permit combination therapies via the co-delivery of multiple therapeutic agents [14,15,16]. Therefore, this review aims to summarize contemporary advanced material design strategies and provide new perspectives for targeted scar therapy.

**Figure 1:**
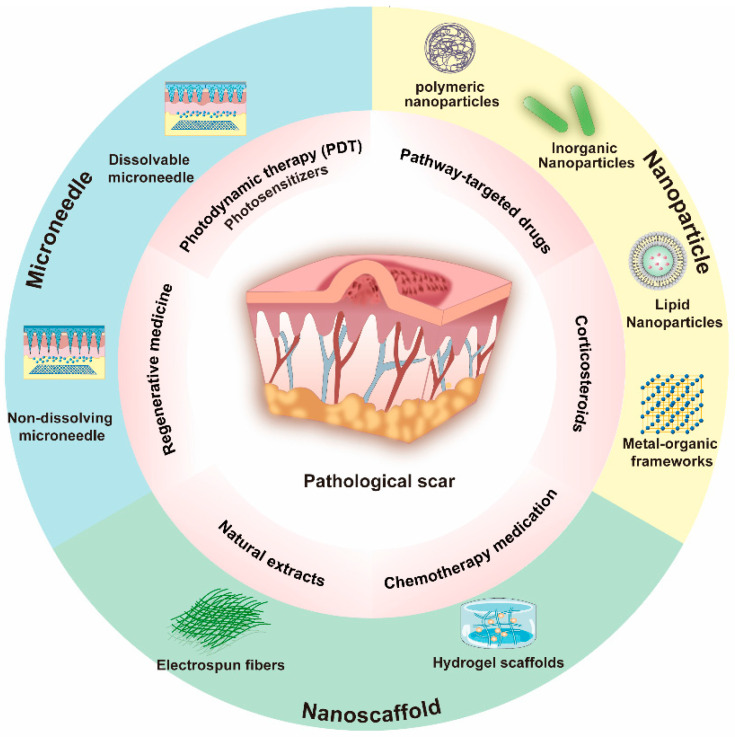
Main therapeutic approaches and drug delivery forms in pathological scar therapy.

This review begins by discussing the pathogenesis of scarring and summarizing the underlying mechanisms of pathological scar development, current therapeutic approaches, and novel biomaterial-based delivery systems (Figure 1). Finally, existing challenges and future directions in scar-targeted delivery systems are addressed.

## 2. Scar Formation and Its Molecular Mechanisms

### 2.1. Wound-Healing Process

The skin serves as the body’s primary protective barrier, and post-injury normal wound healing proceeds through four sequential phases—hemostasis, inflammation, proliferation, and remodeling [4,17,18]. In the hemostatic phase, vasoconstriction and platelet aggregation form a fibrin clot to arrest bleeding [17]; during inflammation, neutrophils and macrophages then clear pathogens and cellular debris to drive the inflammatory response [19]. As healing progresses to the proliferative phase, macrophages switch to an anti-inflammatory phenotype to recruit fibroblasts, which further differentiate into myofibroblasts for extracellular matrix (ECM) synthesis [20,21]. Finally, in the remodeling phase, granulation tissue undergoes apoptosis and matrix metalloproteinase (MMP)-mediated degradation to restore tissue integrity [20].

Pathological scars arise from dysregulated wound healing [1]. Acting as an initiating factor, inflammation triggers an immune cascade, with multiple inflammatory cell types implicated in the development of pathological scars [22,23,24]. Neutrophils release neutrophil extracellular traps (NETs), web-like structures of DNA and cytoplasmic proteins that contain neutrophil elastase and myeloperoxidase [25,26]. These proteases degrade structural proteins, disrupt cell junctions, amplify inflammation, and cause oxidative damage [27]. NETs also sustain the inflammatory milieu by interacting with macrophages, inducing macrophage pyroptosis, promoting a shift toward the pro-inflammatory M1 phenotype, and stimulating the release of tumor necrosis factor-α (TNF-α) and interleukin-6 (IL-6). These cytokines further activate neutrophils, creating a self-perpetuating inflammatory loop [25,28]. Given the central role of NETs in this vicious cycle, targeting NETs has emerged as a cutting-edge therapeutic concept for intervening in pathological scar formation. Potential strategies include suppressing NETosis at its source, for instance, using PAD4 inhibitors to prevent histone citrullination and NET release, as well as clearing preformed NETs by locally applying DNase I to degrade their DNA backbone and dismantle their structure [29,30]. Both approaches hold promise for alleviating NETs-driven tissue damage and fibrotic activation.

**Figure 2:**
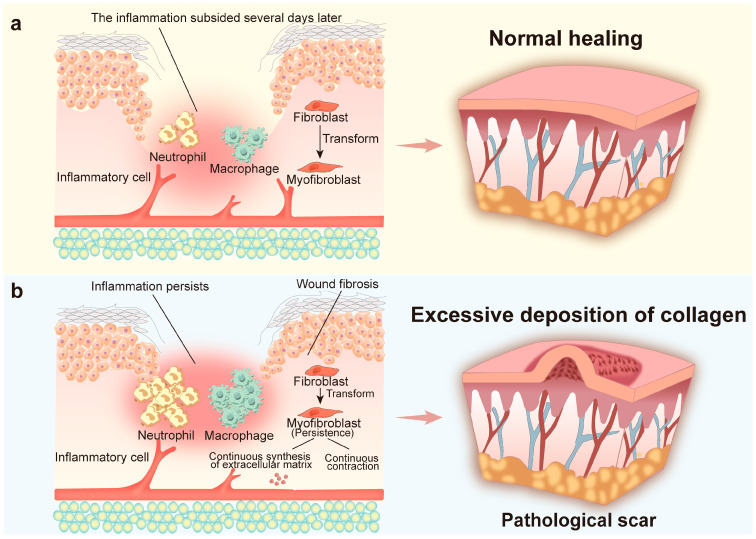
Schematic illustration of the difference between normal wound healing and pathological scar formation. (**a**) Inflammation subsides post-injury; fibroblasts moderately transdifferentiate into myofibroblasts, with no excessive extracellular matrix accumulation, leading to normal skin repair. (**b**): Persistent inflammation lingers at the wound site; myofibroblasts persist here, sustaining contraction while continuously synthesizing extracellular matrix, resulting in excessive collagen deposition and pathological scar formation.

Chronic inflammation disturbs the subsequent proliferative and remodeling phases [31]. In the latter two stages, fibroblasts play a central role. Activated by macrophages and NETs within an inflammatory milieu [26,27], fibroblasts drive abundant ECM deposition and wound fibrosis [32]. The resulting fibrotic matrix generates distinct biomechanical cues that enhance myofibroblast resistance to apoptosis [33]. Consequently, myofibroblasts persist beyond the normal healing period and continue to remodel the tissue. Excess collagen deposition and progressive myofibroblast contraction stiffen the ECM [33]. Aberrant myofibroblast activity further activates M2 macrophages, potentially enhancing arginase-1 release and collagen precursor production, which alters ECM quality [34,35]. This remodeling phase can persist for years and culminates in pathological scar formation [36]. These distinct processes, which include the orderly progression of normal wound healing and the dysregulated development of pathological scarring, are visually illustrated in Figure 2.

### 2.2. Related Signaling Pathways Within Scar Formation

Pathological scarring is driven by the concerted action of multiple signaling pathways—including Transforming Growth Factor-beta (TGF-β), Wnt/β-catenin, Mitogen-Activated Protein Kinase (MAPK), Hypoxia-Inducible Factor 1 (HIF-1), Janus Kinase/Signal Transducer and Activator of Transcription (JAK/STAT), Phosphatidylinositol 3-Kinase/Protein Kinase B (PI3K/AKT), Yes-associated protein/Transcriptional co-activator with PSD-95/Dlg/ZO-1 (PDZ)-binding motif; (YAP/TAZ), and Notch—that promote fibroblast proliferation, collagen accumulation, and persistent inflammation (Table 1). The TGF-β-based axis is central to fibrosis and comprises three isoforms: TGF-β1, TGF-β2, and TGF-β3 [37]. TGF-β1 and TGF-β2 activate the Smad2/3-Smad4 complex, which cooperates with DNA-binding transcription factors to upregulate collagen synthesis and fibroblast activation [38,39]. TGF-β receptors also trigger non-canonical and Smad-independent signaling that cooperates with PI3K/AKT and MAPK to amplify fibrosis [40,41]. The Wnt/β-catenin pathway represents another key axis that interacts with TGF-β signaling to promote the transcription of fibrotic genes [42]. As the core mediator of Wnt signaling, β-catenin can be activated by TGF-β1 and subsequently partners with TCF/LEF transcription factors to drive the expression of target genes [1,43]. On the other hand, the Wnt3a ligand itself induces TGF-β and collagen I production in fibroblasts [44]. Furthermore, scar tissue is often characterized by hypoxia. Local ischemia and the resulting hypoxia stabilize HIF-1α, which upregulates angiogenic and fibrogenic genes and accelerates collagen deposition [45,46]; in the same low-oxygen environment, the PI3K/AKT pathway is simultaneously engaged, heightening glycolysis to drive further fibroblast proliferation and differentiation [47]. The overexpression of pro-inflammatory cytokines, including IL-6 and IL-10, activates the JAK/STAT cascade, thereby enhancing collagen synthesis and fibroblast proliferation [48,49,50,51]. Notch signaling amplifies inflammation [52,53,54], whereas the YAP/TAZ pathway drives fibroblast migration and inhibits apoptosis, enabling the persistence of cells that would normally undergo cell death [53]. The YAP/TAZ pathway is highly mechanosensitive, responding to the mechanical microenvironment of the tissue, which critically regulates scar development [55,56,57].

Therefore, scar formation represents the net outcome of a web-like crosstalk among these pathways, suggesting that combined interventions targeting multiple signaling axes may be a key direction for future anti-scar therapy.

## 3. Scar Treatment Modalities

Pathological scarring risk is primarily determined by wound depth; dermal injury and prolonged healing increase this risk [84]. Epithelialization delay over 10 days or total healing time >3 weeks significantly raises hypertrophic scar chance [84]. Given their adverse impact on quality of life and management difficulties, prevention is critical. Post-trauma, thorough cleansing, debridement and hemostasis reduce infection risk [85]; subsequent care may include pressure therapy, silicone gel, UV protection, etc. [85,86,87]. Nevertheless, these measures do not reliably prevent scarring [4].

Once formed, pathological scars are assessed with standardized tools: the widely used VSS evaluates vascularity, pigmentation, pliability and height [88], while the Asian-tailored JSS includes symptoms and functional impact for long-term follow-up [89]. Additionally, ultrasound (color/power Doppler) provides non-invasive morphological and blood flow data to complement subjective assessments [90]. Based on the assessment, targeted treatments are formulated, including drugs (e.g., corticosteroids), regenerative medicine, photodynamic therapy and surgery. This section reviews these strategies to inform precision scar management.

### 3.1. Invasive Treatment

Among invasive treatments for pathological scars, surgical treatment, laser therapy, and cryotherapy are the main modalities. Surgical excision is a conventional approach, yielding good results for small or linear scars [4]; however, simple excision of keloids carries a high recurrence rate, necessitating adjuvant therapy. Specifically, postoperative radiation can reduce the recurrence rate but poses potential carcinogenic risks, while immediate postoperative injection of triamcinolone acetonide (TAA) serves as a more effective alternative [91,92,93]. Another common invasive modality is laser therapy, which has been utilized for over two decades, with wavelength selection being critical for efficacy [93]. The 585 nm and 595 nm pulsed-dye lasers (PDL) target neovessels to reduce inflammation and improve scar symptoms, though they may cause postoperative purpura [91,94]. In contrast, fractional CO_2_ laser alone has a high recurrence rate and thus requires adjuvant treatment, and its combination with immediate intralesional TAA not only lowers the recurrence rate but also minimizes corticosteroid-related side effects [95,96]. In terms of cryotherapy, it typically uses liquid nitrogen to destroy scar tissue and suppress inflammation [95]; traditional external application has high recurrence rates and numerous side effects due to inadequate deep dermal penetration [97]. Intralesional cryoneedle insertion or −79 °C spray cryotherapy is more effective, though the latter is ineffective as monotherapy and must be combined with TAA or botulinum toxin A [98,99].

### 3.2. Drug Therapy

#### 3.2.1. Pathway-Targeted Drugs

Multiple drugs targeting key signaling pathways offer potential strategies for managing pathological scarring. Among these, The TGF-β pathway serves as the primary target for clinical intervention in scar management. For example, the neutralizing monoclonal antibody fresolimumab has shown potential in clinical trials for improving skin fibrosis, while the small-molecule inhibitor galunisertib has demonstrated anti-scarring effects in preliminary studies and a favorable safety profile in phase II trials for other diseases [100,101,102]. As an important synergistic pathway, the Wnt/β-catenin pathway promotes fibroblast proliferation and epithelial–mesenchymal transition. The CK1α agonist pyrvinium, an FDA-approved drug, facilitates β-catenin degradation, offering a practical route for drug repurposing [103]. Meanwhile, inhibitors of the MAPK pathway—a key node integrating multiple signals—such as trametinib and sorafenib, have been shown to reduce scar formation by suppressing fibroblast activation and collagen deposition [104,105]. Furthermore, inhibitors of the YAP/TAZ pathway, such as verteporfin, not only induce fibroblast apoptosis in animal models but also contribute to the restoration of skin architecture, highlighting their unique therapeutic potential [106]. In contrast, modulators of the HIF-1α, JAK/STAT, Notch and PI3K/AKT pathways hold auxiliary value. HIF-1α modulators (e.g., resveratrol) and JAK/STAT inhibitors (e.g., ruxolitinib) act mainly by regulating hypoxic or inflammatory microenvironments, exerting indirect effects on the core fibrotic process [107,108,109,110]. Notch pathway modulators (e.g., DAPT) also target inflammation and cellular differentiation [111]. The pan-PI3K inhibitor LY294002 can suppress both fibroblast proliferation and activation, but its lack of specificity and clinical validation limits its current applicability [112]. In summary, while the systematic evaluation and clinical translation of these modulators in scar treatment require further development, their repurposing and continued investigation offer a promising prospect for advancing anti-scar therapies.

#### 3.2.2. Corticosteroids

Intralesional corticosteroid injection (ICI) is a standard treatment for pathological scars [113,114,115]. Its mechanism involves dampening inflammation, inhibiting fibroblast proliferation, and promoting collagen degradation [4,114,115]. The most commonly used agent is TAA, typically administered at concentrations of 10–40 mg/mL, with a monthly dose not to exceed 20 mg [115]. A meta-analysis by Zhuang et al. indicated that TAA monotherapy provides short-term benefits, but its long-term efficacy is not superior to that of verapamil, bleomycin, or 5-fluorouracil (5-FU) [116]. Higher concentrations (e.g., 20 mg/mL or 40 mg/mL) increase the risk of skin atrophy and telangiectasia. In a randomized trial, Farrukh et al. reported that 35% of patients experienced adverse effects and 39% relapsed following TAA treatment—rates considered unacceptable by many clinicians [117]. Given the adverse effects of TAA, safer alternatives are being investigated. A double-blind randomized trial found vitamin D as effective as TAA against keloids, with a superior safety profile—notably fewer cases of hypopigmentation and skin atrophy [118]. Combining pentoxifylline (PTX) with TAA also reduces TAA-related side effects and treatment frequency. PTX acts as a vasodilator and hemorheologic agent, improving microcirculation, tissue elasticity, and oxygen delivery to ischemic scar tissue. This enhances scar perfusion and texture while promoting TAA distribution and efficacy within the lesion [119]. As the injections can be painful, lidocaine is often co-administered to mitigate discomfort [113]. Early intervention with tape or plaster splinting can soften the scar and reduce pain associated with subsequent injections [4].

#### 3.2.3. Chemotherapy Drugs

Several chemotherapeutic agents can inhibit scar progression, Their mechanisms of action include inducing fibroblast apoptosis and altering extracellular matrix protein synthesis [120]. The most commonly used agent in this class is 5-FU. As a pyrimidine analog, it inhibits thymidylate synthase, halts DNA synthesis, and suppresses fibroblast proliferation [120,121]. In a study by Gupta et al., lesions were injected weekly with 50–150 mg of 5-FU [122]. Approximately half of the patients achieved greater than 50% keloid flattening, while one-third attained greater than 70% flattening [122]. A meta-analysis by King et al. demonstrated that 67% of patients improved by at least 50%, with a relapse rate of 16% after 27 weeks of follow-up [123]. Collectively, these data establish 5-FU as a recognized treatment option for pathological scars. However, side effects are frequently observed. Saha et al. reported that 95% of patients experienced severe pain during injection, 6% developed superficial ulceration, and others exhibited marked hyperpigmentation [124].

#### 3.2.4. Plant-Derived Active Ingredients

Many phytochemicals are known to accelerate wound healing and promote tissue repair [125]. Bioactive compounds such as phenols, terpenes, and flavonoids can modulate scar-related signaling pathways, exerting effects like promoting apoptosis, inhibiting fibroblast invasion, and suppressing angiogenesis [126]. Representative examples include asiaticoside (a terpenoid) and curcumin (a phenol): asiaticoside inhibits fibroblast proliferation and collagen expression by reducing TGF-β receptor levels, upregulating inhibitory Smad7, and disrupting both the TGF-β cascade and GDF-9/MAPK/Smad axis [127,128]; curcumin acts in a concentration-dependent manner to suppress fibroblast activity, induce apoptosis, regulate scar collagen deposition and inflammation (with efficacy in rabbit ear scar models), and dose- and time-dependently block TGF-β1-induced Smad2 phosphorylation [129,130]. However, research on these phytochemicals remains limited: clinical and in vivo data are scarce, plant sources are heterogeneous, active ingredients are difficult to isolate, and their precise mechanisms of action remain unclear. Table 2 lists the most studied plant products by compound class.

### 3.3. Regenerative Medicine

Regenerative medicine, a pivotal discipline dedicated to restoring tissue and organ function via biological and engineering strategies for repairing damaged structures, serves as a low-adverse-effect modality for scar management [143,144]. Common therapeutic approaches in this field include fat grafting, platelet-rich plasma (PRP), stromal vascular fraction (SVF), and cell therapy. Cell therapy stands out as the most promising approach among these, and its derived products exhibit substantial potential for scar repair: clinically, adipose-derived stem cells (ADSCs) promote cutaneous regeneration and ameliorate scar morphology [145], while allogeneic mesenchymal stem cells (MSCs) reduce the proportion of open wounds in patients with severe burns from approximately one-third to less than 3% in the short term and suppress scar formation [146]. Stem cell-conditioned medium (SCM), a cell-free derivative enriched in bioactive mediators (e.g., growth factors, cytokines, extracellular vesicles), exerts anti-scar effects through targeted molecular mechanisms: adipose-derived SCM (ADSC-CM) inhibits keloid fibroblast proliferation and induces apoptosis by activating the arachidonic acid–COX-2/PGE2 signaling cascade [147,148]; ADSC-derived exosomes attenuate scarring via suppression of the TGF-β1/Smad and PI3K/AKT/mTOR pathways [149,150]; mesenchymal stem cell-conditioned medium (MSC-CM) triggers fibroblast apoptosis in vitro, elicits anti-inflammatory responses, and improves scar outcomes without adverse events in clinical trials [151,152,153,154], thereby emerging as a promising candidate for scar therapy.

### 3.4. Photodynamic Therapy

Photodynamic therapy (PDT) is a treatment modality that involves the combination of a photosensitizer, oxygen, and light [155]. It is widely used in oncology and has applications in dermatology, where it can be delivered using lasers, intense pulsed light, light-emitting diodes (LEDs), or daylight [155,156]. Commonly used photosensitizers include 5-aminolevulinic acid (5-ALA) and its methyl ester, methyl aminolevulinate (MAL) [156]. Both are prodrugs that are metabolized via the heme biosynthesis pathway to generate the endogenous photosensitizer protoporphyrin IX (PpIX) [156]. Upon irradiation, PpIX reacts with tissue oxygen to generate cytotoxic reactive oxygen species (ROS), which selectively target rapidly proliferating cells [156]. PDT is now being explored for the treatment of pathological scars. In vitro studies have shown that PDT induces fibroblast apoptosis [157,158]. When 5-ALA is used, PDT generates mitochondrial ROS, promotes autophagy-dependent cell death rather than classical apoptosis, and downregulates SIRT1 to suppress the SIRT3-SOD2 axis, thereby enhancing its efficacy [159]. Clinically, post-excision 5-ALA-PDT (3–5 sessions) has been shown to prevent auricular keloid recurrence for up to 2.7 years [160]. When combined with a mini-punch technique, it improved ≥50% of lesions in a cohort of 30 patients, reducing Vancouver Scar Scale scores from 9.6 ± 1.1 to 4.2 ± 2.1 [161]. Thus, PDT represents a feasible and promising modality for scar management.

However, PDT still faces significant bottlenecks in clinical application, with the core issue lying in the inherent limitations of traditional photosensitizers. On the one hand, traditional photosensitizers exhibit non-specific photoactivation, which easily triggers off-target toxicity [162]. The resulting cutaneous phototoxicity is the most common complication in PDT treatment, seriously compromising treatment safety and patient tolerability [162]. On the other hand, traditional photosensitizers (e.g., 5-ALA) have poor transdermal penetration ability, failing to cross the dense scar tissue barrier to reach effective therapeutic concentrations, which greatly impairs PDT efficacy [163]. Harnessing drug delivery systems to target and localize photosensitizers at lesion sites while facilitating their penetration across tissue barriers may effectively address these aforementioned limitations of PDT.

## 4. Biomaterial Delivery Systems in Scar Therapy

Conventional invasive approaches, such as surgery and intralesional injections, are often limited by patient discomfort and high rates of recurrence. Biomaterial-based delivery systems address these limitations by enabling targeted transport, sustained release, and enhanced drug stability [164,165]. These systems are now demonstrating significant translational potential. Representative systems, detailed in Table 3, include nanoparticles and microneedles.

### 4.1. Biomaterial Delivery Systems for Drug Delivery

#### 4.1.1. Biomaterial Delivery Systems for Pathway Targeted Drugs

Biomaterial-based drug delivery systems represent a key strategy to overcome pharmacological barriers in scar therapy. The suitability of this strategy varies somewhat across different signaling pathways, depending on their core pathogenic role and the accessibility of corresponding inhibitors. For instance, delivery systems targeting inhibitors of the TGF-β, YAP/TAZ, and PI3K/AKT pathways have been relatively well explored. This is attributable not only to their well-defined roles in pathogenesis but also to the fact that their inhibitors can be readily incorporated into various carriers (e.g., hydrogels, nanoparticles, microneedles) and designed to synergize with specific pathological microenvironments, such as hypoxia or altered mechanical stress, thereby demonstrating higher translational feasibility. In contrast, research on localized delivery systems for MAPK, JAK/STAT, and Notch pathway inhibitors remains notably limited. This gap may arise from insufficient target-specific evidence of these inhibitors in scar pathology or from the fact that relevant studies have largely focused on other disease areas and have not been systematically translated to the field of scar therapy. The following sections will elaborate on the specific delivery strategies and research progress for each of these pathways.

(1) Biomaterial Delivery Systems for TGF-β Pathway Targeted Drugs

The efficacy of locally applied TGF-β pathway inhibitors can be limited by suboptimal drug distribution, short half-life, and inadequate local concentration. Consequently, the development of localized delivery systems for TGF-β-targeting drugs represents a promising therapeutic strategy. For example, Li et al. developed an injectable, self-assembling LA-peptide hydrogel [179]. This gel self-assembles into a nanofiber mesh through β-sheet stacking, facilitating sustained peptide release and gradual degradation. The released LA peptide binds TGF-β with high affinity, which buffers the cytokine level within the wound microenvironment. This action suppresses both PI3K/Akt and TGF-β/Smad2/3 signaling in fibroblasts, ultimately promoting scarless healing. In vivo studies demonstrated that the LA-peptide hydrogel accelerated wound closure and significantly reduced scar formation. A limitation of this study is that the molecular basis for the LA-peptide’s binding to TGF-β was not elucidated.

**Figure 3:**
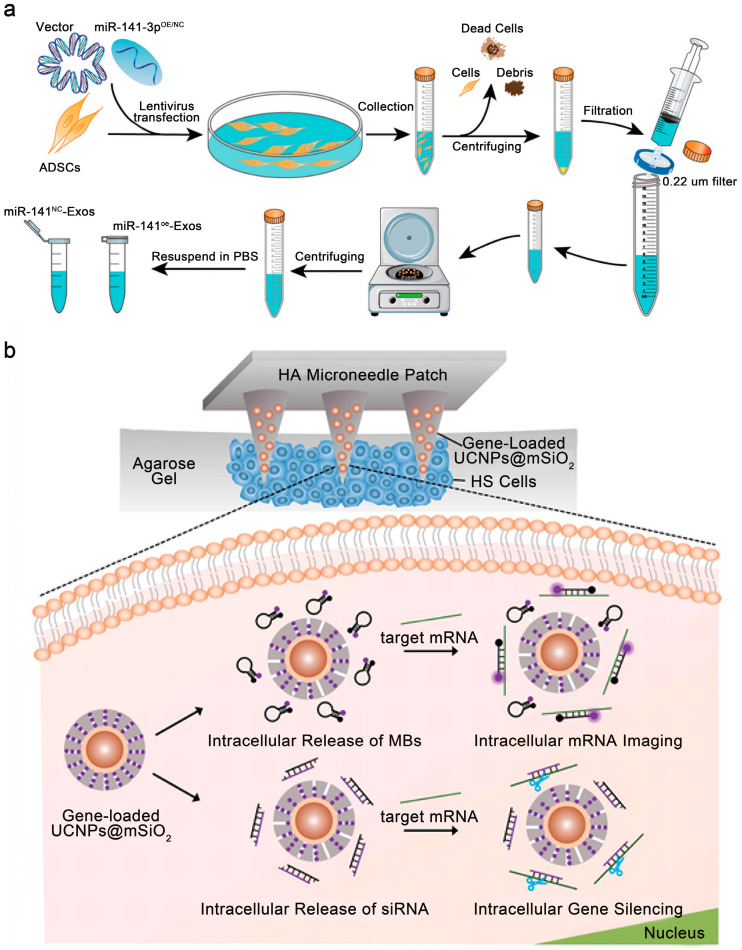
(**a**) Preparation of engineered exosomes loaded with miR-141-3p. Adapted and reproduced, with permission, from references [180]. (**b**) Application of a novel transdermal delivery platform integrating upconversion nanofluorophores (UCNFs) with dissolvable microneedles. Adapted and reproduced, with permission, from references [181].

Furthermore, nanoparticle-based delivery of TGF-β-targeting small RNAs is an area of active investigation. Meng et al. identified miR-141-3p as a scar-suppressive microRNA that inhibits fibroblast proliferation and differentiation [180]. By directly targeting TGF-β2, it interrupts the TGF-β2/Smad signaling axis. To leverage this finding, the same group constructed a dissolving microneedle array (DMNA) composed of hyaluronic acid and hydroxypropyl-β-cyclodextrin to deliver miR-141-3p–enriched engineered exosomes (Figure 3a). In a related approach for siRNA delivery, Wang et al. developed a dissolving microneedle patch incorporating upconversion nanoparticles (UCNPs) for the transdermal administration of TGF-β receptor I–targeting siRNA (Figure 3b) [181]. Upon exposure to near-infrared light, the UCNPs emit detectable luminescence, enabling the mapping of penetration depth and permitting simultaneous treatment and real-time imaging.

(2) Biomaterial Delivery Systems for Wnt/β-Catenin Pathway Targeted Drugs

Fan et al. developed a thermosensitive polypeptide hydrogel for the delivery of the Wnt/β-catenin inhibitor ICG-001 for keloid therapy [182]. The hydrogel degrades into neutral amino acids, ensuring good biosafety. Its thermosensitivity triggers rapid gelation at body temperature and provides sustained ICG-001 release. The released inhibitor induces apoptosis in keloid fibroblasts and demonstrates significant efficacy in vivo. Ahn et al. demonstrated that a non-replicating adenovirus expressing the Wnt decoy receptor sLRP6E1E2 inhibits Wnt signaling and exerts anti-fibrotic effects [183]. The same vector also promotes extracellular matrix degradation in human dermal fibroblasts and primary keloid spheroids. However, transgene expression from the adenoviral vector declines within weeks, necessitating repeated administration. To address this limitation, Yang et al. encapsulated the virus in an alginate gel, which sustains its release and confines the vector to the scar tissue [184].

(3) Biomaterial Delivery Systems for HIF-1α Pathway Targeted Drugs

Resveratrol is an inhibitor of the HIF-1α pathway but faces clinical translation challenges due to its inherent instability and rapid in vivo degradation [185]. Zuo et al. developed mesoporous silica nanoparticles for the encapsulation of resveratrol, improving its solubility and enabling sustained release [186]. Separately, resveratrol has been incorporated into peptide hydrogels to enable in situ release at the wound site, thereby inhibiting scar formation [187]. Huang et al. developed a mechanically skin-like, waterproof, and self-healing bio-elastomer for resveratrol delivery [188]. The drug is loaded through a reversible swelling process that preserves its bioactivity. The material matches the mechanical properties of skin, self-heals underwater, and provides uniform tensile support to counteract wound tension, thereby ensuring precise wound apposition and scar suppression. To date, the delivery of other HIF-1α inhibitors for scar therapy has not been explored. Future work should focus on expanding this delivery strategy to include other HIF-1α inhibitors, thereby broadening the therapeutic arsenal.

(4) Biomaterial Delivery Systems for YAP/TAZ Pathway Targeted Drugs

**Figure 4:**
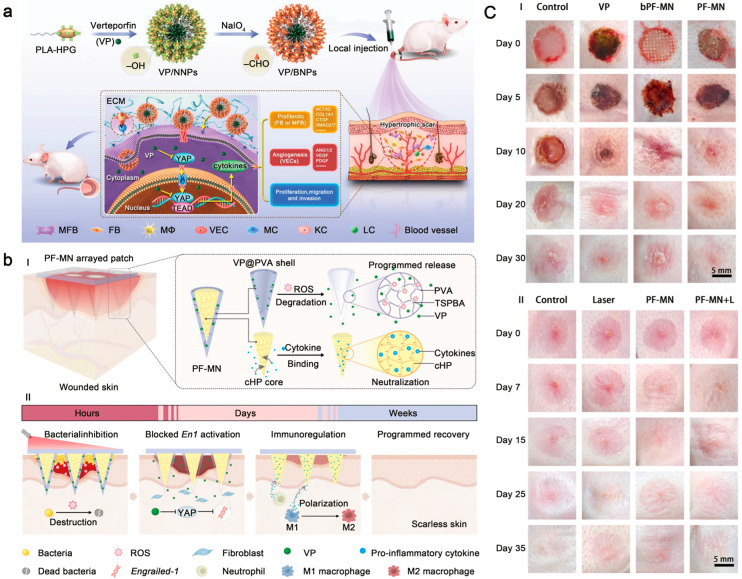
(**a**) Preparation of verteporfin-loaded bioadhesive nanoparticles (VP/BNPs) and their application for treating hypertrophic scars in a rat tail model, where each “×” symbol denotes an inhibitory effect, including suppression of YAP nuclear translocation, YAP-TEAD binding, cytokine production, and the subsequent activation of downstream profibrotic, angiogenic, and cell proliferation/migration/invasion pathways, respectively. Adapted and reproduced, with permission, from references [189]. (**b**) Structure of the core–shell programmable microneedle (PF-MN) and its mechanism for the sequential treatment of chronic wounds. Adapted and reproduced, with permission, from references [190]. (**c**) In vivo evaluation of the PF-MN system for promoting scarless wound healing in a rabbit ear hypertrophic scar model. Adapted and reproduced, with permission, from references [190].

Verteporfin (VP), a potent inhibitor of YAP/TAZ signaling, is considered a promising agent for scar treatment. However, its dose-dependent toxicity to normal cells limits systemic administration [189]. Therefore, targeted delivery of VP to scar tissue is a practical approach to minimize off-target effects. Wang et al. developed bioadhesive nanoparticles (BNPs) for VP encapsulation [189]. The surface hydroxyl groups of the BNPs oxidize to aldehydes, enabling the formation of covalent Schiff-base bonds with tissue amines. This reaction anchors the particles at the lesion, localizing VP and providing sustained release (Figure 4a).

Microneedles also serve as a reliable platform for sustained drug delivery. Zhang et al. fabricated a core–shell microneedle array (PF-MN) designed for the programmed release of stage-specific drugs, which accelerates wound closure and prevents scar formation (Figure 4b) [190]. The microneedle shell is composed of a ROS-sensitive material loaded with a photosensitizer. Upon laser irradiation, ROS are generated, disrupting bacterial biofilms for a potent antibacterial effect. As ROS accumulate, the shell gradually degrades and exposes the core. The core contains cross-linked heparin, which neutralizes pro-inflammatory cytokines such as TNF-α and IL-6. This process curbs excessive inflammation and accelerates the transition from the inflammatory to the proliferative phase of healing. Following shell erosion, VP is released into the deep dermal tissue, where it interrupts mechanotransduction. In a rabbit ear hypertrophic scar model, this sustained-release VP system significantly reduced scar elevation (Figure 4c).

(5) Biomaterial Delivery Systems for PI3K/AKT Pathway Targeted Drugs

**Figure 5:**
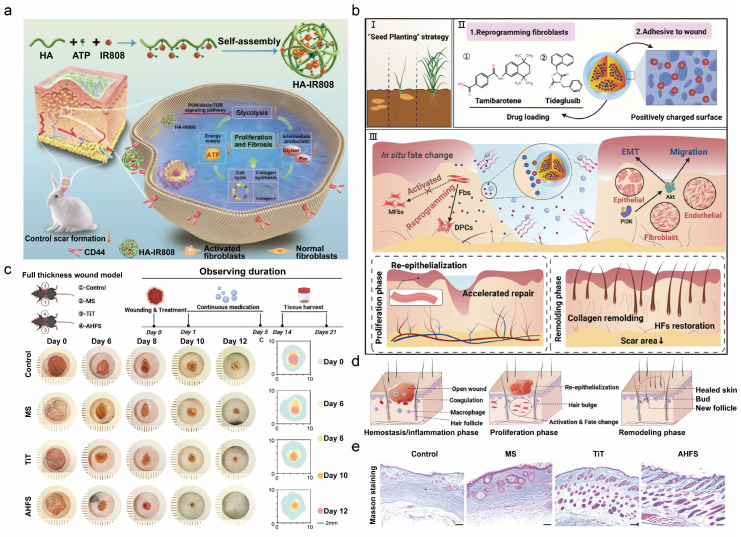
(**a**) Mechanism of the IR808-loaded nanogel in scar prevention via PI3K/Akt/mTOR-mediated glycolysis inhibition to suppress fibroblast proliferation and fibrosis, where all downward arrows denote reductions or inhibitory effects, including decreases in control scar formation, glycolysis, energy supply, proliferation and fibrosis, intermediate production, and Collagen I synthesis. Adapted and reproduced, with permission, from references [191]. (**b**) Multifunctional role of Artificial Hair-Follicle Seeds (AHFS) in promoting wound healing, scarless repair, and in situ hair follicle regeneration through fibroblast-to-dermal papilla cell (DPC) reprogramming, where the “×” symbol in Section III denotes the inhibition of Fbs activation into MFbs. Adapted and reproduced, with permission, from references [192]. (**c**) In vivo therapeutic efficacy and quantitative analysis of AHFS on wound repair. Adapted and reproduced, with permission, from references [192]. (**d**) Process of in vivo fibroblast-to-DPC lineage reprogramming and subsequent hair follicle regeneration. Adapted and reproduced, with permission, from references [192]. (**e**) Masson’s trichrome staining of regenerated skin, demonstrating mature hair follicle structures and collagen deposition, where collagen fibers are stained blue, while cellular components including muscle fibers and cytoplasm are stained red/pink. Adapted and reproduced, with permission, from references [192].

The PI3K/AKT signaling pathway is intricately linked to glycolysis. In pathological scars, glycolytic activity is elevated, representing a key disease-driving mechanism [191]. Based on this rationale, Meng et al. developed a self-assembling transdermal nanogel for the delivery of the glycolytic inhibitor IR808 (Figure 5a) [191]. The nanogel incorporates hyaluronic acid, which exhibits a high affinity for the CD44 receptor on fibroblasts, enabling targeted transdermal delivery. The gel suppresses glycolysis through blockade of the PI3K/Akt/mTOR pathway, reducing ATP availability and macromolecular biosynthesis, thereby curbing collagen over-deposition.

Scar formation impairs skin function and results in the loss of appendages such as hair follicles; the induction of follicle regeneration has been shown to suppress scarring [192]. Ji et al. engineered hydrogel microspheres, termed “artificial hair-follicle seeds” (AHFS) [192]. These microspheres reprogram fibroblasts into dermal papilla cells through chemical induction, enabling in situ follicle regeneration and concurrent scar suppression. The AHFS system integrates a liposomal nanocarrier within a photo-responsive hydrogel shell. This system provides sustained co-release of tideglusib and tamibarotene, activates PI3K/AKT signaling, and accelerates wound closure while inducing follicle neogenesis (Figure 5b). In vitro and in vivo studies demonstrated that the AHFS system accelerates wound closure, reduces scar burden, and regenerates functional hair follicles, offering a novel strategy for functional skin repair and scarless healing (Figure 5c,e).

(6) Biomaterial Delivery Systems for other Pathway Targeted Drugs

To date, biomaterial-mediated delivery of inhibitors targeting the MAPK, JAK/STAT, or Notch pathways for scar mitigation has rarely been reported, but successful applications of such strategies in other disease contexts provide valuable translational insights for anti-scar therapy: PLGA/PLA-based nanocrystal-polymer particles loading the MAPK inhibitor PH-797804 have enabled sustained release for osteoarthritis treatment, offering a model for prolonged anti-scarring drug delivery [193]; hierarchical biomimetic scaffolds releasing the JAK inhibitor ruxolitinib have targeted senescent cells, suppressed the senescence-associated secretory phenotype (SASP), and promoted stem cell osteogenic commitment for bone regeneration—since keloids feature a hypoxia-induced pro-senescence milieu with persistent SASP driving chronic inflammation, this scaffold-based approach could be repurposed to target senescent fibroblasts in scars and disrupt pathological SASP-mediated fibrosis [194,195]; PLGA nanoparticles encapsulating a γ-secretase inhibitor have achieved liver-restricted Notch pathway blockade for obesity-linked metabolic disorders, circumventing intestinal toxicity of systemic administration—this localized delivery strategy is highly relevant for scar therapy, as it could enable site-specific, sustained Notch inhibition in scar tissue to suppress fibrosis while minimizing off-target effects [196]. While such scar-focused delivery remains exploratory, its cross-disease translational potential is significant, and future advances in stimulus-responsive carriers and precision release systems are expected to yield safer, more effective anti-scarring therapies.

#### 4.1.2. Biomaterial Delivery Systems for Corticosteroids

**Figure 6:**
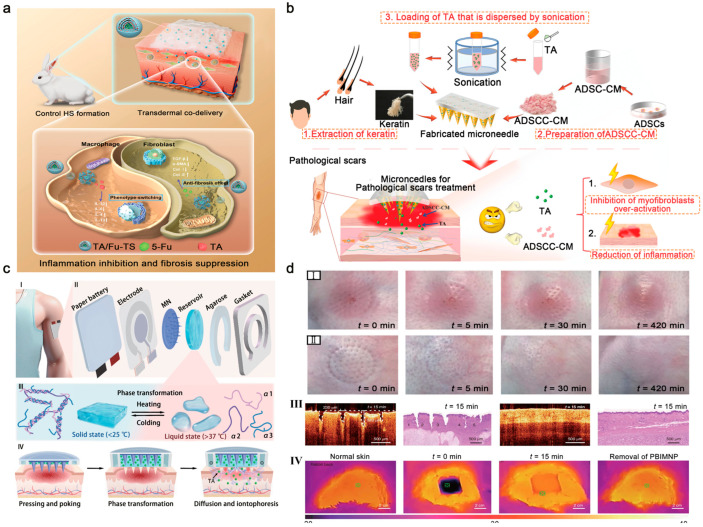
(**a**) Schematic of the transfersome-based nanogel for co-delivery of triamcinolone acetonide (TAA) and 5-fluorouracil (5-FU) in hypertrophic scar therapy, where the downward arrows next to IL-1β, IL-6, α-SMA, TGF-β, and Col I indicate decreases in their respective levels, while the upward arrows next to IL-4, IL-10, and Col III denote increases in their expression. Adapted and reproduced, with permission, from references [197]. (**b**) Preparation and administration of the TA@AC-MN patch. Adapted and reproduced, with permission, from references [198]. (**c**) Composition and administration strategy of the paper-battery-powered iontophoretic microneedle patch (PBIMNP). Adapted and reproduced, with permission, from references [199]. (**d**) Comparative recovery of hypertrophic scars and normal skin following PBIMNP treatment. Adapted and reproduced, with permission, from references [199].

Intralesional injection of TAA is frequently associated with intolerable pain in a subset of patients. The development of advanced delivery systems can reduce injection-related discomfort while achieving more uniform drug distribution. Chen et al. designed a transfersome (TS)-based nanogel for the co-delivery of TAA and 5-FU (Figure 6a) [197]. TS are nanovesicles composed of phospholipid bilayers and an aqueous core, which facilitate the delivery of hydrophobic drugs such as TAA. The co-loading of TAA and 5-FU produces a synergistic anti-inflammatory and anti-fibrotic effect: it suppresses inflammation by promoting macrophage phenotype switching through interleukin-related pathways and inhibits fibrosis by modulating pathways involved in collagen synthesis and degradation.

In addition to nanoparticles and nanogels, microneedle systems have been employed for TAA delivery. Li et al. fabricated a keratin-based microneedle (MN) patch cross-linked with adipose-derived stem cell-conditioned medium (ADSC-CM) for the co-loading of TAA (TA@AC-MN) [198]. This device enabled the sustained co-release of ADSC-CM and TAA (Figure 6b), dampened the inflammatory scar microenvironment, prevented myofibroblast over-activation, and yielded significant scar reduction in a rabbit ear model.

In an innovative approach, Gao et al. developed a paper-battery-powered iontophoretic microneedle patch (PBIMNP) that integrates microneedles with iontophoresis to actively drive TAA into scar tissue (Figure 6c) [199]. The device features a three-layer structure: (1) a drug reservoir module composed of a water-impermeable ring, agarose gel, and gelatin; (2) the temperature-sensitive gelatin, loaded with TAA, enables phase-change-controlled release at 37 °C; The impermeable ring separates the anode and cathode while providing mechanical flexibility, with agarose serving as the anode and gelatin as the cathode for iontophoresis; (3) a microneedle layer that penetrates scar tissue to create microchannels, which, combined with iontophoresis, establishes a dual active-passive drug transport system (Figure 6d); and (4) an iontophoresis module wherein a paper battery linked to a flexible printed circuit board (PCB) supplies a constant current. Notably, the combination of material phase-change properties and self-triggered circuitry maintains a highly integrated structure while enabling the patch to adaptively conform to curved skin surfaces.

#### 4.1.3. Biomaterial Delivery Systems for Chemotherapy Drugs

**Figure 7:**
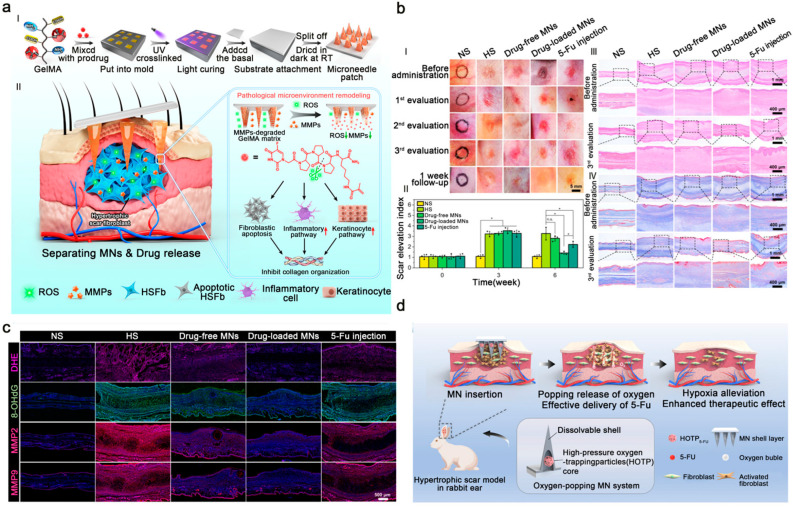
(**a**) Fabrication and therapeutic mechanism of the microneedle (MN) patch for hypertrophic scar treatment, in Section II, the downward arrows next to ROS and MMPs indicate decreases in their respective levels, and the upward arrow next to the keratinocyte pathway and inflammatory pathway denote the activation of these pathways. Adapted and reproduced, with permission, from references [200]. (**b**) Representative scar photographs, Scar Elevation Index (SEI) measurements, and H&E and Masson staining of hypertrophic scars pre- and post-treatment (* *p* < 0.05 and ns indicates no statistically significant difference between groups). Adapted and reproduced, with permission, from references [200]. (**c**) Immunofluorescence staining for reactive oxygen species (DHE, 8-OHdG) and matrix metalloproteinases (MMP-2 and MMP-9). Adapted and reproduced, with permission, from references [200]. (**d**) Schematic of the oxygen-generating MN system for alleviating hypoxia and potentiating 5-fluorouracil (5-FU) efficacy in hypertrophic scars. Adapted and reproduced, with permission, from references [201].

5-FU is widely used for scar treatment; however, intralesional injection is frequently associated with severe pain, hyperpigmentation, and ulceration. Yang et al. employed separable microneedles (SMNs), whose needle tips detach rapidly from the backing to minimize discomfort and enhance patient compliance; additionally, they functionalized a 5-FU prodrug by introducing a methacryloyl group onto its lysine residue [200]. This prodrug was then UV-cross-linked with Gelatin Methacryloyl (GelMA) exclusively in the microneedle tips. The cross-linking density, controlled by the UV dose, allowed for tunable tip strength and drug release kinetics. Furthermore, the MN patch significantly reduces abnormal fibroblast proliferation and collagen deposition, regulates the inflammatory response, and promotes keratinocyte differentiation by upregulating Toll-like receptors (TLRs) (Figure 7a). In vivo, Yang et al. demonstrated that the drug-loaded MNs were more effective than 5-FU injection (Figure 7b). Single-cell sequencing revealed that the MNs scavenge ROS and deplete matrix metalloproteinase-2 and -9 (MMP-2/MMP-9), thereby remodeling the hypertrophic scar microenvironment (Figure 7c).

In contrast to previous strategies, Fu et al. identified intralesional hypoxia as a factor limiting 5-FU efficacy [201]. To counteract this, they developed an oxygen-releasing microneedle system that alleviates local hypoxia and potentiates the efficacy of 5-FU (Figure 7d). This system is a dissolvable core–shell MN platform that encapsulates high-pressure oxygen-trapped particles (HOTPs). In this process, oxygen at 500 psi is injected into a molten carbohydrate mixture and rapidly cooled, thereby trapping the pressurized gas within the solidified matrix. Following application to hypertrophic scars, the pressurized oxygen is rapidly released from the HOTPs, which enhances 5-FU penetration into the dense scar tissue. This microneedle system has demonstrated promising therapeutic outcomes in vivo. Beyond microneedles, 5-FU–loaded nanoparticles have been incorporated into the core of coaxial electrospun nanofibers to create an organic–inorganic hybrid patch [202]. The fibers exhibit favorable wettability, mechanical strength, and degradation profiles, while the sustained release of 5-FU suppresses scar hyperplasia.

#### 4.1.4. Biomaterial Delivery Systems for Plant-Based Natural Products and Extracts

Plant-based natural products and extracts are characterized by their structural diversity and capacity to interact with multiple biological targets. However, their clinical translation is limited by poor stability, rapid metabolism, and a short duration of action [203,204]. Therefore, the development of effective delivery systems is essential to overcome these limitations.

Several researchers have employed hydrogels as carriers for natural products. Lin et al. developed a double-network hydrogel dressing [205]. The primary network consists of a chlorogenic acid (CA)–Zn^2+^ coordination complex. This complex disintegrates within 72 h, releasing CA—a polyphenol from Lonicera japonica with potent antibacterial activity—to address the inflammatory and early proliferative phases of healing. Following the dissociation of the CA–Zn^2+^ bonds, the secondary network, composed of gelatin–heparin, gradually degrades and sustains the release of basic fibroblast growth factor (bFGF) for 30 days, thereby supporting the proliferative and remodeling phases. The embedded CA–Zn^2+^ complex confers antimicrobial, antioxidant, and pro-angiogenic effects, while the sustained release of bFGF suppresses scar formation and accelerates tissue repair. This combination thereby dynamically adapts to the evolving wound microenvironment, representing a programmable delivery strategy for chronic wound and scar management.

**Figure 8:**
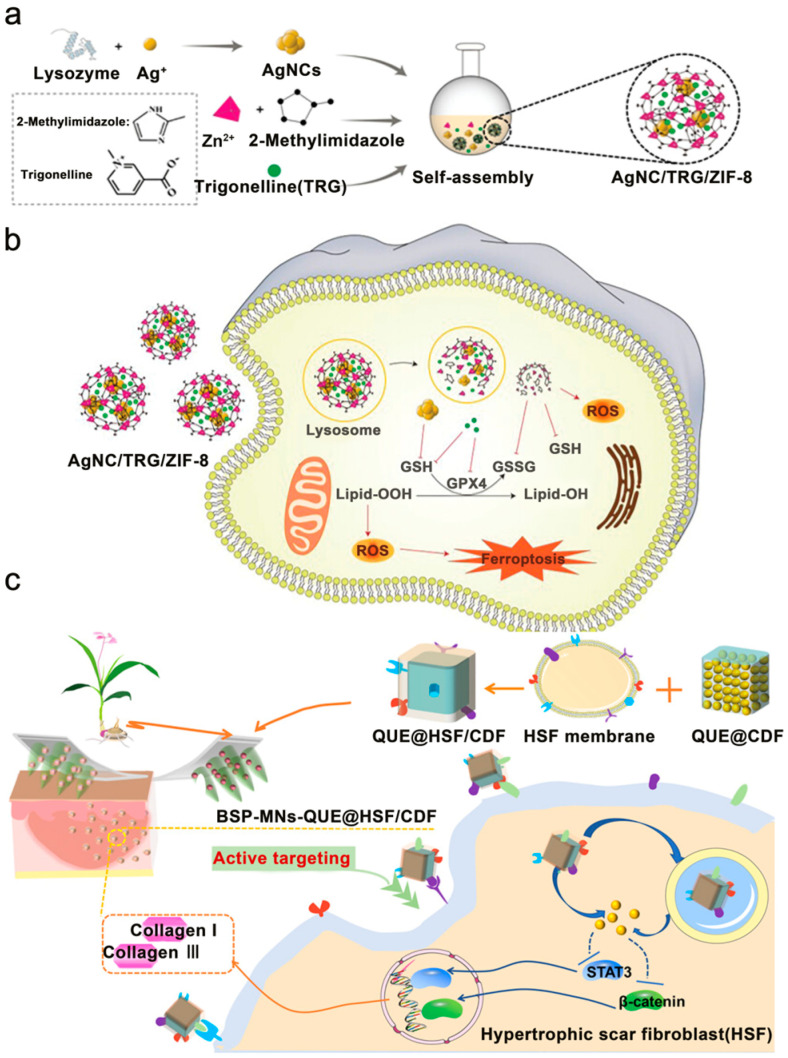
(**a**) Preparation of the AgNC/TRG/ZIF-8 nanoplatform. Adapted and reproduced, with permission, from references [206]. (**b**) Mechanism of AgNC/TRG/ZIF-8-induced ferroptosis in myofibroblasts. Adapted and reproduced, with permission, from references [206]. (**c**) Preparation of the biomimetic transdermal system and its therapeutic effect on collagen I and III deposition. Adapted and reproduced, with permission, from references [207].

Similarly, microneedles have been explored as a delivery platform for natural products and extracts. Zhao et al. identified elevated iron levels in scar tissue and a selective sensitivity of myofibroblasts to ferroptosis. They encapsulated silver nanoclusters (AgNCs) and Trigonella foenum graecum extract (TRG) within a zeolitic imidazolate framework-8 (ZIF-8) to generate an AgNC/TRG/ZIF-8 nanoplatform (Figure 8a) [206]. TRG directly initiates ferroptosis. Upon acid-driven degradation within lysosomes, the released components of the AgNC/TRG/ZIF-8 nanoplatform act synergistically to deplete glutathione, inhibit GPX4 activity, and elevate ROS levels, thereby triggering myofibroblast ferroptosis (Figure 8b). The nanoplatform was incorporated into a GelMAmicroneedle patch, which, when applied to a rabbit ear scar model, significantly improved collagen alignment and reduced scar thickness.

Wu et al. designed a biomimetic transdermal system that targets scar cells (Figure 8c) [207]. Noting that scar-cell membranes exhibit homotypic targeting, they coated quercetin-loaded nanoparticles with these membranes to create biomimetic nanoparticles. These particles were then embedded into dissolvable microneedles for topical application. This targeted delivery system potently inhibited scar-cell proliferation and induced apoptosis, resulting in significant scar attenuation in a rat model.

### 4.2. Biomaterial Delivery Systems for Stem Cells and Conditioned Medium

**Figure 9:**
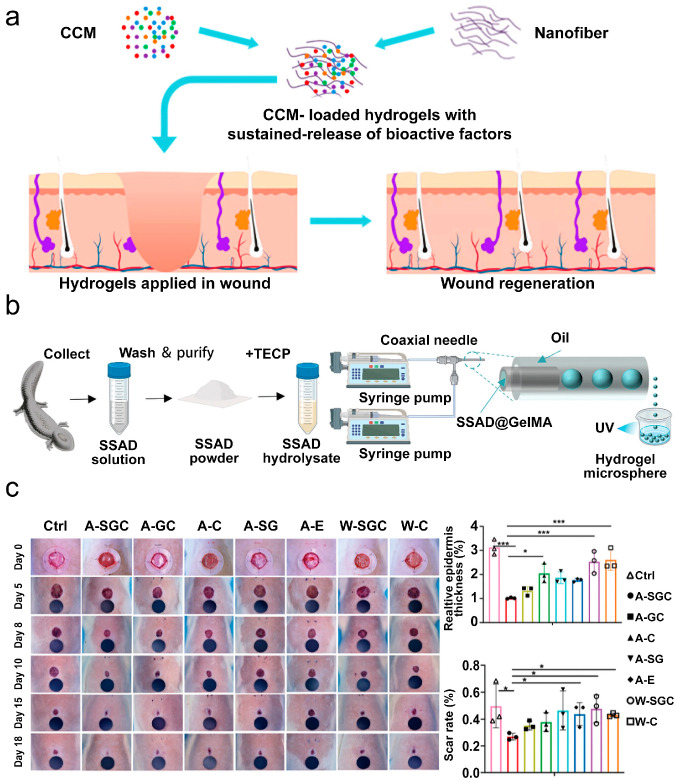
(**a**) Schematic of the mesenchymal stem cell-conditioned medium (MSC-CM) nanofiber composite hydrogel for promoting wound healing and inhibiting scar formation. Adapted and reproduced, with permission, from references [208]. (**b**) Schematic illustrating the preparation of SSAD@GM microspheres. Adapted and reproduced, with permission, from references [209]. (**c**) Representative wound photographs, relative epidermal thickness, and quantitative scar rate analysis in rats from each treatment group at various time points (* *p* < 0.05 and *** *p* < 0.001). Adapted and reproduced, with permission, from references [209].

Mesenchymal stem cell-conditioned medium (MSC-CM) is known to promote tissue regeneration and inhibit scarring. Li et al. encapsulated MSC-CM within a silk fibroin nanofiber-reinforced hydrogel, which preserved its bioactivity and enabled sustained release (Figure 9a) [208]. The sustained release of bioactive factors enhanced fibroblast migration while suppressing their differentiation into myofibroblasts, thereby effectively attenuating scar formation.

Yan et al. pioneered a novel approach combining sustained acupoint stimulation with stem cell therapy [209]. Using a microfluidic technique, they encapsulated human umbilical cord-derived mesenchymal stem cells (hUC-MSCs) within injectable hydrogel microspheres (SSAD@GMs) and administered them into acupoints adjacent to rat wounds (Figure 9b). This acupoint stimulation was found to enhance skin appendage regeneration. Compared to all control groups, the acupoint-injected hUC-MSC-loaded microspheres (A-SGC group) resulted in the most rapid wound closure and the lowest scar incidence (Figure 9c). Multi-omics analyses revealed the activation of amino acid and energy metabolism pathways, concomitant with a reduction in inflammatory mediators. Single-cell RNA sequencing demonstrated a marked expansion of pro-repair cell populations, including M2 macrophages, myofibroblasts, and endothelial cells, confirming the therapeutic mechanism.

Shafiee et al. fabricated a medical-grade polycaprolactone (mPCL) biomimetic dressing via melt electrospinning writing (MEW), a 3D printing technology. Human gingival mesenchymal stem cells (hGMSCs) were integrated into the dressing via cell seeding [210]. Furthermore, the cryopreservation protocol was optimized to ensure the viability and functionality of the hGMSCs within the construct. The aligned microarchitecture of the scaffold enhanced its mechanical strength, promoted cell adhesion and proliferation, accelerated full-thickness wound closure, and reduced scar area in a rat model.

### 4.3. Biomaterial Delivery Systems for Photodynamic Therapy Photosensitizer

Although PDT is employed for scar management, the poor penetration of photosensitizers such as 5-ALA across biological barriers significantly limits its clinical efficacy [211]. Furthermore, inadequate accumulation of 5-ALA within scar tissue can induce protective autophagy in fibroblasts during PDT, thereby diminishing its therapeutic effect [211]. To address these limitations, Huang et al. developed a comprehensive PDT strategy [211]. They fabricated a hyaluronidase (HAase)-based microneedle array co-loaded with 5-ALA and metformin. This array facilitates 5-ALA diffusion and enhances its penetration into the deep dermis. Metformin elevates local oxygen tension and disrupts autophagic homeostasis, thereby potentiating PDT-induced cell death. In vitro and in vivo studies confirmed that this regimen suppresses PDT-induced protective autophagy and significantly reduces the scar elevation index, representing an efficient and minimally invasive anti-scarring modality.

**Figure 10:**
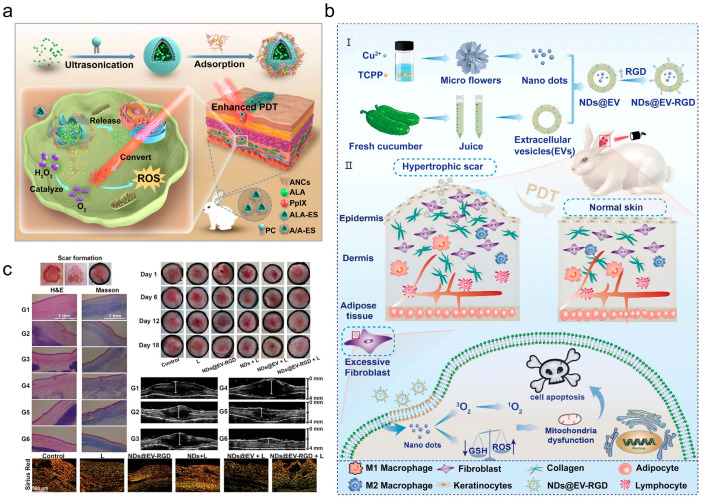
(**a**) Illustration of the A/A-ES nanovesicle and its mechanism of self-generated oxygen production for enhanced PDT efficacy. Adapted and reproduced, with permission, from references [212]. (**b**) Preparation and transdermal delivery of NDs@EV-RGD nanoparticles for targeted photodynamic therapy, in Section II, the downward arrow next to GSH and the upward arrow next to ROS indicate a decrease in GSH concentration and an increase in ROS production, respectively. Adapted and reproduced, with permission, from references [213]. (**c**) In vivo therapeutic efficacy of the NDs@EV-RGD nanoplatform in reducing scar thickness and suppressing collagen deposition via ROS generation and GSH depletion for targeted PDT. Adapted and reproduced, with permission, from references [213].

The efficacy of PDT is limited by the hypoxic core of scar tissue, as PDT requires both a photosensitizer and molecular oxygen. Although oxygen is scarce, scar lesions contain an abundance of endogenous hydrogen peroxide (H_2_O_2_) [212]. Ultrasmall gold nanoclusters (AuNCs) can catalyze the decomposition of H_2_O_2_ to generate oxygen. Capitalizing on this, Chen et al. engineered a functional transdermal nanovesicle by electrostatically immobilizing AuNCs onto the surface of 5-ALA-loaded ethosomes (termed A/A-ES) [212]. The A/A-ES system simultaneously delivers 5-ALA and functions as a catalase-like nanozyme, self-generating oxygen to enhance PDT efficacy, promote scar fibroblast apoptosis, and overcome the intrinsic hypoxia of scar tissue (Figure 10a).

A limitation of 5-ALA is that it must be metabolized in situ to its active porphyrin form prior to light activation. Kong et al. bypassed this metabolic step by incorporating porphyrin directly into a metal–organic framework (MOF), creating a MOF-based photosensitizer that significantly enhances porphyrin-mediated PDT [213]. To overcome the poor skin penetration of large MOFs, the researchers engineered ultrasmall copper-based MOF nanodots (Cu-MOF NDs) and fused them with cucumber-derived extracellular vesicles (EVs) to enhance tissue penetration. Simultaneously, the EVs were conjugated with an arginine-glycine-aspartic acid (RGD) peptide. The RGD peptide confers targeted delivery by binding specifically to αvβ3 integrin on scar fibroblasts, facilitating high accumulation of the NDs@EV-RGD construct at the scar site. Following accumulation at the scar site, near-infrared (NIR) irradiation triggers robust ROS generation and concurrent glutathione (GSH) depletion, effectively inducing fibroblast apoptosis and suppressing collagen deposition (Figure 10b). In vivo, this nanoplatform significantly reduced scar thickness and collagen content, demonstrating strong therapeutic potential (Figure 10c).

## 5. Discussion

Pathological scars, arising from aberrant wound healing, severely impair patients’ quality of life; the dense scar tissue limits drug penetration, leading to suboptimal efficacy and side effects of traditional treatments with little fundamental progress. Biomaterial-based delivery systems (especially nanomaterials and microneedles) have emerged as promising strategies, enabling efficient, minimally invasive skin barrier penetration for precise and sustained drug delivery.

Nevertheless, the clinical translation of these systems faces considerable challenges. First, significant gaps remain in safety and biocompatibility assessments. For instance, the long-term biosafety of inorganic nanoparticles is a major concern [214]. While they can enhance drug delivery, their long-term in vivo distribution, metabolic clearance pathways, and the potential chronic toxicity risks associated with accumulation in organs such as the liver and spleen lack systematic long-term tracking studies [215]. At the application level, repeated use of microneedles may provoke local immune responses, cause micro-injuries to tissues, and produce cumulative effects, which could reduce patient compliance [216]. Additionally, long-term therapeutic efficacy remains challenging. Most current delivery systems focus on suppressing acute fibrotic processes but inadequately address fibroblast “memory” or microenvironmental reprogramming, potentially leading to the reactivation of pathological processes and scar recurrence upon treatment withdrawal. Furthermore, bottlenecks in translating laboratory findings to the clinic are particularly prominent. Beyond scalability issues for platforms such as electrospun scaffolds [217], many advanced delivery systems remain confined to preclinical models, partly due to the complex regulatory pathways associated with combination products (e.g., drug-device combinations like microneedles). The translation process must also fully address regulatory and ethical considerations, including meeting stringent requirements for long-term safety data, establishing robust quality control standards for mass production, and ensuring thorough informed consent, equitable participant selection, and comprehensive risk-benefit assessment in clinical trials. Moreover, the scarcity of multicenter, large-sample clinical trials and confirmatory efficacy evidence further hinders clinical translation. To systematically evaluate and compare the potential of various delivery platforms in addressing these challenges, Table 4 summarizes their therapeutic efficiency, safety profiles, and current status of clinical translation.

To address these hurdles, future research should focus on three key areas: enhancing biosafety evaluation (e.g., by establishing porcine skin models integrated with fluorescence tracking), driving synergistic innovation in materials and processes, and prioritizing multicenter, large-sample clinical trials to obtain confirmatory efficacy evidence while simultaneously addressing the relevant ethical considerations and regulatory issues during clinical translation. Successfully tackling these issues will facilitate the seamless transition of biomaterial-based delivery systems from laboratory research to clinical practice, thereby fostering transformative advances in scar therapy.

## Figures and Tables

**Table 1:** Key molecular signaling pathways involved in scar formation and their crosstalk.

Molecular Pathways	Role in Scar Formation	Crosstalk with Other Pathways	References
TGF-β	Excess TGF-β1 and TGF-β2 drive excessive fibroblast activation and abnormal collagen synthesis and deposition, whereas TGF-β3 exhibits anti-fibrotic activity.	TGF-β interacts with Wnt/β-catenin. It mediates JAK/STAT3 and YAP/TAZ through Smad, and it also cooperates with PI3K/AKT and MAPK in a Smad-independent manner.	[44,58,59,60]
Wnt/β-catenin	Sustained β-catenin activity drives fibroblast migration, proliferation, activation and collagen synthesis.	Besides TGF-β, Wnt signaling may also crosstalk with Notch during wound healing, while PI3K and JNK could act upstream of β-catenin. Further studies are needed to clarify how Wnt interacts with other pathways in scar formation.	[61,62,63,64]
MAPK	It chiefly modulates scar formation through crosstalk with the TGF-β pathway.	MAPK and TGF-β are known to crosstalk. MAPK also modulates Wnt signaling in tumors. Yet reciprocal regulation between MAPK and Wnt in scar formation has not been reported.	[40,65,66]
HIF-1α	It stimulates fibroblast proliferation, suppresses apoptosis, promotes collagen synthesis, and enhances glycolysis under hypoxia.	HIF-1α activates the TGF-β pathway. PI3K/AKT also drives scar formation by up-regulating HIF-1α. Additionally, HIF-1α crosstalks with STAT3 and the ERK/MAPK cascade during scarring.	[45,46,47,67,68,69,70]
JAK/STAT	It promotes collagen synthesis and fibroblast proliferation.	In keloid formation, TGF-β engages STAT3 to stimulate fibroblast proliferation. The non-canonical Wnt ligand Wnt5a may also crosstalk with JAK/STAT, and IL-10 has been reported to facilitate crosstalk between PI3K/AKT and STAT3 signaling.	[48,49,50,51,71,72]
Notch	It amplifies inflammation, increases collagen deposition, reduces autophagic flux in keloid fibroblasts, and enhances their migration and invasion. The same factor can also promote hypertrophic scar formation by skewing keratinocyte differentiation.	Notch crosstalk with TGF-β influences tissue fibrosis, and during wound healing Notch also interacts with Wnt. In defined cellular contexts, YAP/TAZ mediate Notch-driven transcription.	[52,53,54,73,74,75,76]
YAP/TAZ	It enhances cell proliferation, suppresses apoptosis, drives fibroblast-to-myofibroblast conversion, and increases collagen deposition.	YAP/TAZ enhances TGF-β signaling by promoting nuclear translocation of Smad2/3, and Notch forms a positive feedback loop with YAP/TAZ.	[77,78,79,80]
PI3K/AKT	PI3K/AKT, together with other regulators, promotes fibroblast proliferation and migration, suppresses apoptosis, and drives collagen synthesis. Under hypoxia, it enhances glycolysis in keloid fibroblasts and may facilitate their differentiation.	This axis forms a positive feedback loop with HIF-1α under hypoxia and crosstalks with TGF-β, Wnt/β-catenin and JAK/STAT.	[47,81,82,83]

**Table 2:** Plant-derived compounds with anti-scar effects.

Compound Types	Active Ingredients	The Effect of Scar Treatment	Affected Pathways	References
Terpenoids	Asiaticoside	Inhibits fibroblast proliferation and collagen expression.	Reduces TGF-βRI and TGF-βRII levels while raising inhibitory Smad7, thereby disrupting the TGF-β cascade; also blocks the GDF-9/MAPK/Smad axis.	[127,128,131]
Phenols	Curcumin	Suppresses fibroblast proliferation, migration, and activation; promotes apoptosis in a concentration-dependent manner. Modulates collagen deposition and inflammation within scars. Shows good efficacy in rabbit ear scar models. In addition, the curcumin derivative DMC-HA demonstrates superior inhibitory effects on fibroblast proliferation and apoptosis induction in vitro compared to curcumin.	Inhibits TGF-β1-induced Smad2 phosphorylation in a dose- and time-dependent manner.	[129,130]
	Resveratrol	Reverses the pro-proliferative effect of hypoxia on keloid fibroblasts, suppresses collagen synthesis, and promotes apoptosis.	Inhibits the HIF-1α pathway.	[132,133]
Flavonoids	Quercetin	Inhibits fibroblast proliferation and collagen expression in a dose-dependent manner; in vivo, it reduces the number of activated macrophages and myofibroblasts at rabbit ear wound sites and suppresses inflammation.	Blocks TGF-β/Smad signaling and suppresses its downstream targets; completely disrupts the association of Smad2, Smad3, and Smad4.	[134,135,136]
Glycosides	Ginsenoside Rg3	Rg3 inhibits fibroblast proliferation, induces apoptosis, suppresses angiogenesis, and reduces collagen synthesis. It also dampens inflammation and accelerates wound healing.	Inhibits TGF-β/Smad and ERK pathways.	[137,138,139]
Extracts	Onion extract	Onion extract inhibits fibroblast proliferation. Clinical trials show that combining onion extract with silicone gel or its derivatives, or with triamcinolone acetonide, reduces scar color, height, stiffness, and itching.	Block the IGF-I signaling and the TGF-β/Smad signaling pathway in fibroblasts.	[120,140,141,142]

**Table 3:** Key biomaterial carriers for localized drug delivery in scar management.

Types		Properties	Advantages	Applications	References
Nanoparticles	Polymeric nanoparticles	Biodegradable, water-soluble, biocompatible, and biomimetic.	They deliver drugs of varying molecular weights and provide sustained release.	Used in cancer therapy, gene delivery, and diagnostics.	[166,167,168]
	Inorganic nanoparticles	They possess distinct physicochemical and optical properties, high crystallinity, large surface area, and are readily functionalized.	They can be engineered into diverse structures and sizes, and utilized in multiple formats.	Applied in diagnosis, imaging, and photothermal therapy.	[167,168]
	Metal–organic frameworks	High porosity, versatile function, and diverse structure–composition.	Easy to synthesize and functionalize, with high drug-loading and release capacity; encapsulates bioactive molecules and is amenable to X-ray characterization.	Used for drug delivery, biomimetic catalysis, biosensing, photodynamic therapy, and photothermal therapy.	[169,170,171]
	Lipid-based nanoparticles	Low toxicity and high biocompatibility.	Simple formulation, self-assembling, high bioavailability, strong payload capacity.	They have entered the clinic and achieved remarkable success in immunotherapy.	[167,168]
Nanoscaffold	Electrospun fibers	High surface area, microporosity, high drug-loading capacity, and controlled-release properties.	Enables month-long drug delivery, offers high drug loading, mimics natural extracellular matrix architecture, and creates new opportunities for wound healing.	Modulates stem-cell behavior, accelerates cell proliferation, and promotes wound healing.	[172,173]
	Hydrogel scaffolds	Soft, wet materials composed of polymeric chains, fibers, or particles that form a three-dimensional porous network. Their physical and chemical properties can be tuned to accelerate solute diffusion, and some hydrogels exhibit self-healing capacity.	Widely used on diverse wounds and tissues, they maintain a moist microenvironment and prevent infection.	Used as wound dressings, sensors, drug-delivery vehicles, and tissue-engineering scaffolds.	[174,175,176]
Microneedles	Non-dissolving microneedles\Dissolvable microneedles	High mechanical strength; create micron-scale, transient skin channels for drug delivery. \Biocompatible and biodegradable; dissolve in skin for sustained drug release.	Non-invasive; minimize bleeding and infection; enable delivery of high-molecular-weight drugs into skin. easy to manufacture, simple to apply, and well accepted by patients.	Primarily used for drug delivery.	[177,178]

**Table 4:** Comparative evaluation of delivery platforms for scar therapy.

Types		Delivery Performance	Scalability and GMP Compatibility	Safety Profile	Translational Product in Clinical Trial Phase	References
Nanoparticles	Polymeric nanoparticles	Capable of encapsulating drugs of varying molecular weights and enabling their controllable and sustained release.	Traditional wet chemistry methods, such as nanoprecipitation, face scalability bottlenecks including significant batch-to-batch variation, high solvent consumption, and complex purification steps. Dry techniques like plasma polymerization offer a promising alternative, enabling continuous, solvent-free, and scalable manufacturing. While GMP production is feasible for specific polymers like PLGA, it demands stringent process control.	Although biodegradable, they are prone to particle aggregation, which increases toxicity risks.	Few therapeutic products have advanced to clinical trials in areas such as pathological scars and wound healing.	[167,218]
	Inorganic nanoparticles	Possessing unique physical properties and being amenable to functionalization, which confers additional attributes and enhanced delivery capabilities.	Traditional batch synthesis faces challenges of decreased mixing and mass transfer efficiency during scale-up, leading to significant batch-to-batch variations. Continuous production technologies, such as high-gravity reactors, represent a key direction for addressing these scalability issues.	Their low solubility and potential for long-term accumulation pose toxicity concerns, particularly in formulations containing heavy metals.	In the field of wound healing, randomized controlled trials have demonstrated that silver nanoparticles can effectively accelerate the healing process in burn patients and chronic refractory wounds. Furthermore, preliminary case reports indicate that physiological inorganic polyphosphate nanoparticles also exhibit promising therapeutic efficacy in wound healing.	[167,219,220,221,222]
	Metal-organic frameworks	Exhibiting exceptional drug loading capacity due to their ultra-high specific surface area and tunable porosity.	Conventional laboratory-scale methods rarely meet commercial and supply chain demands. Emerging processes such as spray drying, continuous flow reactors, and twin-screw extrusion offer a promising path toward continuous and scalable commercial production.	Toxicity is highly dependent on the administration route and their chemical/colloidal stability in biological media, as these factors influence aggregation and degradation behavior in vivo.	Few therapeutic products have advanced to clinical trials in areas such as pathological scars and wound healing.	[223,224]
	Lipid-based nanoparticles	As liposomes are readily absorbed by the reticuloendothelial system, they are often surface-modified to prolong systemic circulation and improve targeting delivery.	Microfluidic technology enables scalable, GMP-compliant production at a rate of 200 mL/min.	Despite high biocompatibility, their efficacy may be limited by low drug-loading capacity and suboptimal biodistribution, often leading to high uptake in the liver and spleen.	Few therapeutic products have advanced to clinical trials in areas such as pathological scars and wound healing.	[167,225]
Nanoscaffold	Electrospun fibers	The high specific surface area, microporosity, high drug loading capacity, and controlled release performance make electrospun fibers an ideal carrier for controlled release and targeting.	Despite its wide application, conventional electrospinning presents limitations such as weak mechanical strength, broad diameter distribution of the produced nanofibers, and challenges in process scale-up. These shortcomings necessitate the introduction of improved techniques, like reactive electrospinning, to overcome these drawbacks and enhance fiber performance.	Natural polymeric materials exhibit excellent biocompatibility; however, high-voltage operation and solvent residue remain concerns requiring caution.	A clinical study demonstrated that electrospun chitosan nanofiber materials effectively promoted wound healing in patients with type IIIa and IIIb burns. The study included 19 patients with type II and IIIa burns, as well as 10 patients with type IIIb burns.	[226,227]
	Hydrogel scaffolds	Hydrogels exhibit diverse morphologies, enable stimulus responsiveness, and possess high drug loading and controlled release capabilities.	The scalability of natural hydrogels from laboratory to industrial-scale production relies on precise control of key parameters including rheological properties, reactor design and optimization of application technologies, while GMP compliance requires the establishment of comprehensive quality management systems, traceability mechanisms and risk assessment processes to achieve a balance between process standardization and feasibility of clinical translation.	Excellent biocompatibility and tunable degradation properties.	A clinical study demonstrated that a hydrogel/nanosilver dressing promoted the healing of diabetic foot ulcers. The study involved 60 patients with type 2 diabetes who presented with diabetic foot wounds.	[228,229]
Microneedles	Non-dissolving microneedles\Dissolvable microneedles	Non-dissolving microneedles offer high drug loading capacity and tunable release rates, whereas dissolvable microneedles have a drug payload limited by their needle volume, typically resulting in a rapid, burst-release profile.	For scalable production, insoluble microneedles (e.g., silicon, metal) depend on precision techniques like photolithography and laser machining, which are mature but costly to scale; GMP compliance requires strict adherence to regulations, ensuring biocompatibility, process control, and sterility. In contrast, dissolvable microneedles are mainly produced industrially via solvent casting due to its reliability and ease of use, while emerging methods like photolithography are still developing.	Non-dissolving microneedles require aseptic handling and carry a residue risk, whereas dissolvable microneedles offer superior biocompatibility and biosafety.	Dissolvable hyaluronic acid microneedles delivering secreted protein acidic and cysteine-rich (SPARC)-targeting siRNA were shown to reduce scar-side fibrosis in a 30-patient, randomized, single-blind, intra-individual controlled trial.	[230,231,232]

## Data Availability

No datasets were generated or analyzed during the current study.

## Abbreviations

5-ALA, 5-Aminolevulinic Acid; 5-FU, 5-Fluorouracil; ADSCs, Adipose-Derived Stem Cells; ADSC-CM, Adipose-Derived Stem Cell-Conditioned Medium; AuNCs, Gold Nanoclusters; bFGF, Basic Fibroblast Growth Factor; BNPs, Bioadhesive Nanoparticles; CA, Chlorogenic Acid; Cu-MOF NDs, Copper-based Metal-Organic Framework Nanodots; DMNA, Dissolvable Microneedle Array; ECM, Extracellular Matrix; EVs, Extracellular Vesicles; GelMA, Gelatin Methacryloyl; GSH, Glutathione; HAase, Hyaluronidase; HIF-1α, Hypoxia-Inducible Factor-1alpha; HOTPs, High-pressure Oxygen-Trapped Particles; hUC-MSCs, Human Umbilical Cord-derived Mesenchymal Stem Cells; ICI, Intralesional Corticosteroid Injection; IL-6, Interleukin-6; ICG-001, (6S,9aS)-Hexahydro-6-[(4-hydroxyphenyl)methyl]-8-(1-naphthalenylmethyl)-4,7-dioxo-N-(phenylmethyl)-2H-pyrazino[1,2-a]pyrimidine-1(6H)-carboxamide; IR808, 2-(2-(2-((1E,3E,5E,7E,9E,11E)-12-(4-(dimethylamino)phenyl)dodeca-1,3,5,7,9,11-hexaen-1-yl)-3-ethylbenzothiazol-3-ium-2-yl)vinyl)-3-ethylbenzothiazol-3-ium)ethyl sulfate; JAK/STAT, Janus Kinase/Signal Transducer and Activator of Transcription; MAL, Methyl Aminolevulinate; MAPK, Mitogen-Activated Protein Kinase; MMPs, Matrix Metalloproteinases; MOFs, Metal-Organic Frameworks; MNs, Microneedles; MSCs, Mesenchymal Stem Cells; MSC-CM, Mesenchymal Stem Cell-Conditioned Medium; mPCL, Medical-grade Polycaprolactone; MEW, Melt Electrospinning Writing; NETs, Neutrophil Extracellular Traps; NIR, Near-Infrared; NPs, Nanoparticles; PBIMNP, Paper-Battery-Powered Iontophoretic Microneedle Patch; PCB, Printed Circuit Board; PDT, Photodynamic Therapy; PI3K/AKT, Phosphoinositide 3-kinase/Protein Kinase B; PLA, Polylactic acid; PLGA, Poly(lactic-co-glycolic acid); PpIX, Protoporphyrin IX; PRP, Platelet-Rich Plasma; PTX, Pentoxifylline; ROS, Reactive Oxygen Species; SASP, Senescence-Associated Secretory Phenotype; SCM, Stem Cell-Conditioned Medium; SEI, Scar Elevation Index; siRNA, Small Interfering RNA; SMNs, Separable Microneedles; SVF, Stromal Vascular Fraction; TAA, Triamcinolone Acetonide; TGF-β, Transforming Growth Factor-beta; TLRs, Toll-like Receptors; TNF-α, Tumor Necrosis Factor-alpha; TRG, Trigonella foenum graecum extract; TS, Transfersomes; UCNPs, Upconversion Nanoparticles; UV, Ultraviolet; VP, Verteporfin; VSS, Vancouver Scar Scale; YAP/TAZ, Yes-associated protein/Transcriptional co-activator with PSD-95/Dlg/ZO-1 (PDZ)-binding motif; ZIF-8, Zeolitic Imidazolate Framework-8.
